# The Quebec Rural Emergency Department Project: A Cross-Sectional Study of a Potential Two-Pronged Strategy in the Knowledge Transfer Process

**DOI:** 10.1371/journal.pone.0120523

**Published:** 2015-04-07

**Authors:** Mélodie-Anne Drouin, Richard Fleet, Julien Poitras, Patrick Archambault, Jean-Marc Chauny, Jean-Frédéric Lévesque, Mathieu Ouimet, Gilles Dupuis, Alain Tanguay, Geneviève Simard-Racine, Josée Gauthier, Fatoumata Korika Tounkara, Marie-Hélène Gilbert, France Légaré

**Affiliations:** 1 Department of Family and Emergency Medicine, Université Laval; Québec, QC, Canada; 2 Research Chair in Emergency Medicine Laval University—CHAU Hôtel-Dieu de Lévis Hospital; Lévis City, QC, Canada; 3 Department of Family and Emergency Medicine, Université de Montréal; Department of Emergency Medicine, Hôpital du Sacré-Coeur de Montréal, QC, Canada; 4 Centre for Primary Health Care and Equity of the University of New South Wales, New South Wales, Australia; 5 Département de science politique, Pavillon Charles-De Koninck, Université Laval, Québec, QC, Canada; 6 Département de psychologie, Université du Québec à Montréal, Montréal, QC, Canada; 7 Departement of Emergency Medicine, CSSS de La Matapédia, Québec, QC, Canada; 8 Direction de l’analyse et de l’évaluation des systèmes de soins et services, Institut national de santé publique du Québec, Université du Québec à Rimouski, Rimouski, QC, Canada; 9 Department of Family Medicine and Emergency Medicine, Knowledge Transfer and Health Technology Assessment of the CHUQ Research Centre (CRCHUQ), Unité de Recherche Évaluative, Université Laval, Québec, QC, Canada; Community Information Epidemiological Technologies, CANADA

## Abstract

**Introduction:**

Health services research generates useful knowledge. Promotion of implementation of this knowledge in medical practice is essential. Prior to initiation of a major study on rural emergency departments (EDs), we deployed two knowledge transfer strategies designed to generate interest and engagement from potential knowledge users. The objective of this paper was to review: 1) a combined project launch and media press release strategy, and 2) a pre-study survey designed to survey potential knowledge users’ opinions on the proposed study variables.

**Materials and Methods:**

We evaluated the impact of the project launch (presentation at two conferences hosted by key stakeholders) and media press release via a survey of participants/stakeholders and by calculating the number of media interview requests and reports generated. We used a pre-study survey to collect potential key stakeholder’ opinions on the study variables.

**Results:**

Twenty-one of Quebec’s 26 rural EDs participated in the pre-study survey (81% participation rate). The press release about the study generated 51 press articles and 20 media request for interviews, and contributed to public awareness of a major rural research initiative. In the pre-study survey, thirteen participants (46%) mentioned prior knowledge of the research project. Results from the pre-study survey revealed that all of the potential study variables were considered to be relevant for inclusion in the research project. Respondents also proposed additional variables of interest, including factors promoting retention of human resources.

**Conclusions:**

The present study demonstrated the potential utility of a two-pronged knowledge transfer strategy, including a combined formal launch and press release, and a pre-study survey designed to ensure that the included variables were of interest to participants and stakeholders.

## Introduction

With over 14 million patient visits each year, emergency departments (EDs) represent an important component of the public health care system in Canada [1]. However, the majority of studies in emergency medicine are conducted in academic centers located in urban areas [2], and little information concerning rural EDs is available. The practice of emergency medicine in rural areas differs significantly from practice in urban centers, and research in this area has identified challenges specific to rural practice [3–6]. Considerable seasonal fluctuation in patient volume in EDs [7], difficulty accessing training for specialized life-saving procedures and for continuing medical education [8], physician shortages, and lack of consultant support [9, 10] constitute only a few examples of the challenges facing rural practice.

Research on rural emergency care is critical, yet faces considerable challenges [11, 12]. Most rural EDs are not university-affiliated, and prior staff experience in research participation is often limited. Challenges inherent to work in rural settings can combine with busy clinical schedules due to staffing shortages to discourage participation in research. Moreover, involvement in research, particularly in the field of health care, may be limited by unfamiliarity with the research process, lack of opportunity to participate in research projects, and limited understanding of statistical analyses [13, 14]. Promoting participation in research in rural settings requires creative dissemination strategies. The scientific community must contribute to the development of knowledge tailored to the realities and needs of rural EDs, and explore novel strategies to support evidence-based decision-making.

Our multidisciplinary research group is conducting a major descriptive and evaluative study of rural EDs in Quebec [15]. To the best of our knowledge, ours is the first large-scale research study on rural EDs in Canada. As such, it constitutes a unique opportunity to develop a solid foundation for further knowledge transfer and participant engagement by involving potential knowledge users in the initial phases of the project. This paper reports on two strategies that were developed to meet this objective. The first strategy involved a combined project launch and media press release; the objective of this strategy was to publicize the project and to optimize the participation rate. The second strategy involved obtaining knowledge users’ opinions concerning study variables before the research project was initiated. Finally, we sought to estimate level of familiarity among rural hospital staff with the provincial ED management Guide (published in 2006 by the Quebec’s Ministry of Health and Social Services to help improve performance among Quebec’s EDs) [16]. The guide contains recommendations specific to rural EDs and could serve as a reference for establishing standards of emergency care across Canada [16].

## Materials and Methods

This cross-sectional study is based on data collected from knowledge users. Inclusion criteria for EDs required 24/7 medical coverage and in-patient hospitalization beds. Local community service centers, mobile care units, and private clinics were therefore excluded. Rural EDs (N = 26) were identified using the Guide to Canadian Health Care Facilities and were confirmed by the Quebec’s Ministry of Health and Social Services and the province’s “*Direction nationale des urgences”* [17]. A rural area was defined according to Statistics Canada criteria [18]. To participate in the study, emergency physicians, emergency clinician managers, and hospital directors had to be working for at least one of the 26 rural EDs in Quebec selected for this study.

### Strategy 1: Combined project launch and media press release strategy

Strategy 1 involved a combined project launch and media press release. First, the primary investigator (RF) launched the project at the annual meetings of two key provincial stakeholders meetings: the “*Association des gestionnaires infirmiers d’urgence du Québec* (AGIUQ)” [Quebec Emergency Nurse Manager’s Association] in May 2011 (approximately 200 attendees), and the “*Association des médecins d’urgence du Québec* (AMUQ)” [Quebec Emergency Physicians’ Association] in October 2011 (approximately 1000 attendees). The two launches involved formal presentation of the study, and answering stakeholder questions about the project at a conference information booth. Second, concurrently with the AMUQ meeting, a single press release announcing the launch of the research project was sent to major newspapers and specialized media, as well as to regional and rural media. The newsletter was released by the Laval University Department of information and communications.

To evaluate the reach of the press release and the two presentations, the pre-study survey (further described below) contained the following questions: “Had you heard about this research project before you were asked to complete this form? If so, how?” The pre-study survey was sent to participants one month after the project launch and media press release; impact was measured using two independent media monitoring firms.

### Strategy 2: Pre-study survey

The pre-study survey consisted of a structured online and paper questionnaire. The paper version was designed to be distributed to emergency physicians attending the October 2011 annual AMUQ meeting. The electronic version was subsequently created using the software program Survey Monkey (California, USA); it included the majority of the questions in the paper version, and was sent by email to potential respondents.

### Examples of questionnaire items

1. Name the three most significant challenges in providing rural emergency care that research should address on a priority basis.
2. Indicate your level of familiarity with the recommendations in the provincial EDs management Guide [16] on a scale of 1 to 4, ranging from “not at all” to “very” (paper questionnaire), and on a scale of 1 to 7 ranging from “not at all” to “extremely” (online questionnaire).
3. Propose factors that should be investigated in order to facilitate the implementation of the recommendations in the provincial EDs management Guide [16] in rural EDs.
4. Rate the perceived importance of the following proposed variables for the larger study: characteristics of the population served, geographical and roadway characteristics, distance between hospital and other specialized services, upstream and downstream resources, ambulance services, air services, hospital characteristics, medical personnel and outpatient clinic, computer support for medical decision-making, knowledge transfer activities, ED visits, trauma data, diagnostic services, number of ED nurses, number of emergency physicians, other professionals and employees, use of the provincial EDs management Guide, performance and quality-of-care indicators, and ED personnel quality of work-life.
5. Suggest further variables that should be examined in order to accurately describe Quebec’s rural EDs.

Participants were given examples to facilitate understanding of questionnaire items. They were asked to respond on a scale ranging from 1 (“not important at all”) to 5 (“essential”) for the paper version of the questionnaire, and on a scale from 1 “not useful at all” to 7 (“extremely useful”) for the online version.

Rural emergency physicians attending the October 2011 annual meeting of the AMUQ were invited to participate in the paper version of the pre-study survey. We also solicited online opinions from key health-care policy-makers, including representatives from medical and nursing associations and federations (e.g., AMUQ, AGIUQ, Quebec College of Physicians, Quebec’s Ministry of Health and Social Services, Quebec’s health and social services agencies, advisory groups [e.g., the TCCAT], university emergency medicine program directors, and medical school deans). Regular and part-time emergency physicians, two types of emergency clinician managers (ED chiefs and head nurses) and three types of hospital directors (CEOs, directors of professional services, and directors of nursing) from each of Quebec’s rural EDs were also invited to participate in the online survey.

### Data analysis

For strategy 1, we assessed the potential impact of this strategy by calculating degree of participant knowledge about the study, using data collected in the pre-study survey within one month of the launch and press release. The impact of the media press release strategy was further assessed by two independent media monitoring firms and reported by Laval University’s Department of information and communications. For the purpose of this study, we tallied the total number of interview requests and media reports about the study.

The pre-study survey (strategy 2) contained several open-ended questions concerning variables to be included in the research project, challenges faced by emergency medicine in rural areas, and obstacles to applying the provincial ED management Guide. To analyze the qualitative data, each raw response was placed in a more general category to reduce the number of possible responses. Two research assistants verified the classification into categories to ensure validity. To identify the most frequently cited and most significant challenges, we compared the frequency of citing for each category. To identify overall trends and specific trends according to employment category, we calculated means and standard deviations for all participants and for each employment group (emergency physicians, emergency clinician managers, hospital directors, and policy-makers). In contrast with the 5-point measurement scale used in the paper version of the questionnaire (administered first), the electronic version used a 7-point scale. Because data from the two questionnaires were not initially meant to be analyzed together, we used two different measurement scales (1 to 4 and 1 to 7). To consolidate the data across scales, we used the cross-multiplication procedure [19], which allowed us to analyze all of the data on a scale from 1 to 7. We also transposed all of the answers onto a scale from 1 to 4. To complete our consolidation, we conducted a sensitivity analysis, that is, an investigation of the potential changes and errors between the two strategies, and their impact on the study conclusions [20].

### Ethical considerations

The CSSS Alphonse-Desjardins Research Ethics Committee approved the pre-study survey. The objectives and procedures were explained to each participant, and participant anonymity was respected at all levels of the procedure.

## Results

### Strategy 1: Combined project launch and media press release strategy

Twenty-eight participants (two emergency physicians, five emergency clinician managers, 18 hospital administrators, and three policy-makers) responded to the question about prior knowledge of the research project (“Had you heard about this research project before you were invited to complete this form?”). Of the 28 participants respondents, thirteen answered “yes” (46%) and 15 answered “no” (54%). Of those who responded “yes,” 8% reported that they had heard about the study at the meeting at Laval University, 15% had heard about it from a third party, and 23% heard about it from the investigators (data not shown). Of the 13 participants who had heard about the project prior to the survey, seven (54%) named a source of information related to the combined media strategy (“media” or “AGIUQ”).

The press release generated 51 press articles and 20 media requests for interviews. Furthermore, staff from four of Quebec’s 26 rural EDs spontaneously contacted the lead investigator for more information.

### Strategy 2: Pre-study survey

Forty-three participants completed either the paper or electronic version of the pre-study survey. Seven emergency physicians completed the paper version, and two emergency physicians, seven emergency clinician managers, 18 hospital directors, and nine policy-makers completed the online version. Overall, 81% of Quebec’s rural EDs contributed data to at least one questionnaire. As for the online questionnaire, the response rate was 14% for emergency clinician managers, 24% for hospital directors and 50% for policy-makers. Since emergency physicians had been approached via a booth at the AMUQ meeting, their response rate could not be determined.

When participants were asked to rate the importance of each potential study variable, diagnostic services was deemed to be the most useful variable (see Table 1). In descending order, the next four highly rated variables were distance between hospital and other specialized services, ED visits, computer support, and number of ED nurses. The variable ranked least important was air services, although mean score remained high at 4.81. All variables were therefore considered useful for the upcoming research project.

**Table 1:** Participant ranking (in decreasing order) of importance of study variables on a scale from 1 “not at all useful” to 7 “extremely useful”.

Variable	Mean ± SD^*^
Diagnostic services	6.31 ± 0.99
Distance between hospital and other specialized services	6.29 ± 0.87
Emergency department visits	6.21 ± 1.01
Computer support	6.16 ± 0.96
Number of nurses in the emergency department	6.09 ± 1.04
Number of emergency physicians	6.08 ± 1.09
Medical personnel and outpatient clinic	6.06 ± 1.14
Ambulance services	6.05 ± 0.94
Upstream and downstream resources	5.99 ± 1.22
Performance/quality-of-care indicators	5.92 ± 1.06
Hospital characteristics	5.84 ± 1.06
Other professionals and employees	5.83 ± 1.07
Knowledge transfer	5.77 ± 1.03
Quality of work-life	5.66 ± 1.37
Characteristics of the population served	5.52 ± 1.13
Trauma	5.33 ± 1.32
Use of the QEMDG	5.33 ± 1.17
Geographical and roadway characteristics	5.27 ± 1.35
Air services	4.81 ± 2.01

^*^SD = Standard Deviation

Participants were also asked to identify additional variables that should be examined (see Table 2). In light of the results, we added certain variables to the larger study, including factors promoting retention of human resources. The measurement instruments were also adapted to better meet participant needs.

**Table 2:** New variables for inclusion proposed by participants.

Variables
Existing staff experience (age, years of service, work experience, training, career plan)
Factors that promote personnel retention
Available specialized medical equipment
Geographical location of the health-care facility
Physical layout of the premises
Medical and nursing man-power plans and the functions of medical and nursing personnel
Contribution of regular and occasional locum physicians
Maintenance of competence and teaching
Employee and emergency physician work satisfaction
First-line medical services: care intensity, visits, priorities 1 to 5, alternative measures in the event of overcrowding, advanced interventions requiring ABCD
Rural hospitals’ performance
Impact of following the QEDMG
Regional orientation/availability of funds (organization)
Availability of database information
Service interruptions in the past few years

We also collected data concerning the challenges of providing rural emergency care. Results are presented in Table 3. The most frequently cited challenge across participants was lack of human resources/access to specialized services, followed closely by maintenance of competence/training of medical and nursing personnel. Opinions varied across the four groups of participants, although there was some overlap between answer categories, and some similarities were identified.

**Table 3:** Frequency of citing for challenges faced by emergency medicine in rural Quebec (by entire sample and by job category).

Challenges faced by emergency medicine in rural areas	All participants	Emergency physicians^*^	Clinician managers^$^	Policy-makers^¶^	Directors^€^
Lack of human resources/access to specialized services	40	4	7	9	20
Maintenance of competence/training of medical and nursing personnel	36	6	4	11	15
PESs/interhospital transfers (access to and distance between specialized centres)/maintaining service corridors	23	6	5	4	8
Access to technical facilities and diagnostic services	15	4	2	5	4
Upstream and downstream resources	15	─	3	1	11
Organization of services	4	─	1	2	1
Personnel recruitment and retention	4	─	─	1	3
Emergency department length of stay/overcrowding	3	─	1	─	2
Integration of locum physicians	3	─	─	─	3
Lack of medical protocols/standardization of practices	3	1	─	─	2
Computer support/telehealth	2	─	─	─	2
Access to new drugs	1	1	─	─	─
Physical layout of the emergency department	1	─	1	─	─
Funding	1	─	─	─	1
Variation in number of visits	1	─	─	─	1
Administrative “red tape”	1	─	─	─	1

* Emergency physicians: nine regular or locum emergency physicians

^$^Clinician managers: seven emergency clinician managers, specifically, emergency department chiefs and head nurses

^¶^Policymakers: nine health-care policy-makers who did not work in hospitals

^€^Directors: 18 directors at rural Quebec hospitals: specifically, CEOs, directors of professional services, and directors of nursing

Participants were asked to indicate their level of familiarity with the recommendations in the provincial ED management Guide (data not shown). The mean obtained across all groups was 3.7; in general, participants were partially familiar with the guide’s recommendations. Standard deviation was 2.0, which indicates considerable differences between participants. Standard deviation decreased when each group’s data was analyzed separately. Contrary to expectations, emergency physicians were the group that was the least familiar with the guide, with a mean of 1.2. Emergency clinician managers were the most familiar with the guide, with a mean of 5.0. Policy-makers’ familiarity with the guide (4.2) was similar to the level of familiarity among hospital directors (4.2).

The responses concerning potential barriers to implementing the guide’s recommendations are presented in Table 4. For the sample as a whole, maintenance of competence/training of medical and nursing personnel emerged was the most frequently mentioned barrier. Barriers cited differed between the four groups.

**Table 4:** Frequency of citing for factors that impede the use of the provincial ED management Guide, by entire sample and by job category.

Factors that impede the use of theProvincial ED management Guide	All participants	Emergency physicians^*^	Clinician managers^$^	Policy-makers^¶^	Directors^€^
Maintenance of competence/training of medical and nursing personnel	14	4	5	1	4
PESs/Interhospital transfers (access to and distance between specialized centres)/maintaining service corridors	10	5	2	1	2
Lack of human resources/access to specialized services	10	3	1	2	4
Upstream and downstream resources	10	1	1	3	5
Access to technical facilities and diagnostic services	7	2	─	1	4
Dissemination of guide recommendations (physicians, nurses, managers, and directors)	7	─	2	2	3
Non-medical personnel expertise	6	─	1	─	5
Team composition	5	─	─	─	5
Adapting the guide	3	─	─	2	1
Funding	3	─	1	─	2
Bed management	3	─	1	1	1
Computerization of emergency departments	3	─	─	1	2
Organization of services	3	─	─	1	2
Triage quality	3	1	─	─	2
Personnel recruitment and retention	3	─	1	2	─
Physical layout of the emergency department	2	1	─	─	1
Lack of human resources/access to specialized services/funding	2	─	─	1	1
Screening tools	2	─	─	─	2
Regional hospital support/consultation at the regional level	2	1	1	─	─
Mandatory application	1	─	1	─	─

* Emergency physicians: nine regular or locum emergency physicians

^$^Clinician managers: seven emergency department clinician managers (emergency department chiefs and head nurses

^¶^Policymakers: nine health-care policymakers who did not work in hospitals

^€^Directors: 18 directors at hospitals in rural Quebec (CEOs, directors of professional services, and directors of nursing)

## Discussion

### Strategy 1: Combined project launch and media press release strategy

The combined project launch and media press release strategy appears to have successfully informed the target audience of the project, as evidenced by the percentage of participants in the pre-survey study who reported prior knowledge of the study (46%). Of these participants, 54% identified a source of information related to the strategy (“media” or “AGIUQ” conference). Furthermore, the press release generated 51 press articles and 20 media requests for interviews. Given the relatively small number of news outlets in rural Quebec, the press release was considered to have a strong impact among the media. Four of Quebec’s rural EDs contacted the second author (RF) for further information before the research project began.

Several studies [21, 22] have demonstrated the potential benefit of using mass media as a strategy for disseminating research results and thereby changing health behaviors. Mass media dissemination is a key step in the knowledge transfer process, but few data are available on the effectiveness of this strategy for involving knowledge users at an early stage of a project. The perceived importance of research by its users predicts data use [23–25] and knowledge transfer [26]. Although it will be difficult to quantify, we anticipate that the combined project launch and media press release strategy will foster interest, engagement, and participation in the larger study.

The strategy implemented here was inexpensive; incurred costs primarily concerned the time invested in the launch and in the media interviews. However, the impact of a press release is unpredictable. Media coverage depends on perceived general public interest for a given subject at a particular point in time. Media coverage also depends on competing current events. Based on the present report, we recommend the implementation of our combined strategy (formal study launch and press release) particularly when attempting to generate interest from potential participants and stakeholders who are otherwise difficult to reach and to engage.

### Strategy 2: Pre-study survey

Surveying participants and stakeholders about their perceptions of what is relevant and important to study in their clinical settings is a key knowledge transfer strategy, and one that is often neglected. It demonstrates investigators’ interest in participant issues, and helps to confirm and improve study variables.

In the pre-study survey phase, we found that participants considered all of the planned study variables to be relevant, with diagnostic services ranked as the highest priority for study. The additional suggested variables were used to improve the research project. The primary reported challenge of providing rural emergency care was lack of human resources/access to specialized services. Participants were partially familiar with the provincial ED management Guide, although familiarity among emergency physicians was limited. The primary barrier to the application of the guide was maintenance of competence/training of medical and nursing personnel.

Given that different types of participants (policy-makers, director, clinician managers, and emergency physicians) had different concerns and different interests in the project, the pre-study survey results were examined according to type of participant. Each group has a different role and a different level of decision-making power [26], and the same data will therefore be used differently across groups of participants. For example, research results can be transformed into a decision-making tool for policy-makers and into an intervention grid for health professionals [26]. It is therefore critical to have a strong understanding of target audience when undertaking a research project. To that end, the pre-study survey was designed to confirm relevant factors and to identify new variables deemed important by participants. For instance, researchers and policy-makers do not operate in the same spheres of activity and therefore do not have quite the same needs or constraints [27]. It is therefore critical to include knowledge users’ opinions when determining research goals and priorities prior to initiating a research project. In our project, the variables deemed to be of greatest interest by the investigators concerned the characteristics of the region, hospitals and EDs, and prehospital services and inter-facility transfers. However, participants also cited additional variables of interest, including personnel recruitment and retention factors. In sum, our results suggest that a pre-study survey addressing participant concerns is effective to improve the research protocol. Further, engaging stakeholders from the onset is likely to promote participation in the overall study.

### Strengths and limitations

This study has certain limitations. First, participation rate varied according to type of respondent, and may not be entirely representative of Quebec’s rural EDs and health care policy-makers. Further reminders for participation may have optimized the participation rate, but were prevented by time constraints.

Second, it was difficult to assess the precise impact of the press release. There is presently no consensus among researchers regarding the optimal method for assessing the impact of a mass media knowledge transfer strategy [26–28]. While two independent media monitoring firms gauged the impact of our press release and presentations by calculating the number of interviews and articles generated, we do not know how well this measure fostered actual participation in the study of Quebec’s rural emergency departments. Certainly, the pre-study survey suggested an increased awareness of the project, and designing a press release with the help of communications specialists may be an easy and inexpensive knowledge transfer strategy.

Despite these limitations, the pre-study survey had a number of strengths. First, it addressed issues that have not been extensively studied in the literature. The results can therefore serve as a basis for future studies of rural hospitals and mass media knowledge transfer strategies. Second, we presented the results for the group as a whole, and also for each different employment group (emergency physicians, emergency clinician managers, hospital directors, and policy-makers). The conclusions of this study are therefore applicable to very specific groups. We also modified variables according to the respective group interests. Furthermore, we clearly defined the term “rural” at the outset of the study, and used rigorous methodology for identifying Quebec’s rural emergency departments. Finally, the results of the pre-study survey reflect the opinions of participants from a large proportion (81%) of Quebec’s rural EDs.

## Conclusions

This study demonstrated the potential usefulness of a two-pronged knowledge transfer strategy that included a combined formal study launch and press release, and a pre-study survey that helped us modify study variables to meet the informational needs of participants and stakeholders. This study therefore constitutes a starting point for the development of new integrated knowledge transfer strategies.
